# Economic uncertainty and mental health: Global evidence, 1991 to 2019

**DOI:** 10.1016/j.ssmph.2024.101691

**Published:** 2024-06-14

**Authors:** Emre Sarı, Buse Şencan Karakuş, Ender Demir

**Affiliations:** a School of Business and Economics, UiT the Arctic University of Norway, Tromsø, Norway; b Division for Health and Social Sciences, NORCE Norwegian Research Centre, Oslo, Norway; c Division of Developmental Pediatrics, Department of Pediatrics, Hacettepe University Faculty of Medicine, Ankara, Turkiye; d Department of Business Administration, School of Social Sciences, Reykjavik University, Reykjavik, Iceland

**Keywords:** Economic uncertainty, Anxiety disorders, Major depressive disorder, Eating disorders, Public health policy, Mental health

## Abstract

Mental health has deteriorated globally due to COVID-19, climate crisis, economic policies, and regional conflicts, requiring immediate attention. This study aims to comprehend the relationship between economic uncertainty and the prevalence of anxiety disorders, major depressive disorder, and eating disorders across various demographics and countries. Using robust fixed-effect models, we analyzed the relationship between economic uncertainty and mental disorders in 110 countries from 1991 to 2019. Our analysis also explored whether this association varies across genders and age groups. Our analysis indicates that economic uncertainty is associated with higher prevalence rates of anxiety and major depressive disorders, though no similar association is observed for eating disorders. In the subgroup analyses, while females have a significant association exclusively with anxiety disorders, males have associations with anxiety and major depressive disorders. The age-specific analyses show that economic uncertainty is associated with anxiety disorders for almost all age groups above 15 years, except for ages between 40 and 54. For major depressive disorders, this association becomes significant after the 40–44 age group. However, we see no significant association among age groups for eating disorders.

## Highlights

• Globally, economic uncertainty is associated with higher mental disorder prevalence.
• Anxiety disorders and major depressive disorder differ among age cohorts.
• Economic uncertainty is associated with higher anxiety disorders among females.

## 1 Introduction

As we navigate the complexities of the 21st century, we find ourselves confronting a silent yet pervasive crisis—mental health. Now a global challenge, it affects nearly 12.5% of people worldwide as of 2019 (Global Burden of Disease Collaborative Network, 2020). Further, surveys up to 2022 show that by age 75, about half of individuals are likely to have experienced a mental disorder (McGrath et al., 2023). Mental disorders, such as anxiety and major depressive disorder, are recognized not only as individual challenges but also as global challenges, with an alarming increase in the incidence of mental illness worldwide. Each country struggles to address the issue of professional resources, available facilities, and economic burdens. The COVID-19 pandemic has amplified this struggle, intertwining with the climate emergency, escalating humanitarian issues, fluctuating economic policies, and ongoing regional conflicts (Kola, 2020; Kola et al., 2021). Kola et al. (2021) highlight the significant disruption to life brought on by the COVID-19 pandemic, signaling a critical need for more attention to mental health services during such global crises.

Understanding the economic uncertainty becomes essential in the current global mental health landscape (Er et al., 2023; Liu, 2023). Massazza et al.’s (2023) scoping review reveals a consistent positive correlation between uncertainty and mental health issues, highlighting the need for more diverse and robust research to understand and address this relationship. Complementing this, recent studies by Er et al. (2023), Claveria (2022), and Vandoros et al. (2019) corroborate the link between economic uncertainty and global suicide trends. Parallel research exploring economic uncertainty in relation to subjective health (Tao & Cheng, 2023), and cardiovascular disease mortality (Kawachi et al., 2023) also show significant associations, suggesting mental disorders as a potential underlying mechanism. Despite these findings, conclusive evidence on the relationship between economic uncertainty and mental disorders, particularly anxiety disorders, major depressive disorder, and eating disorders, at a broader scale has yet to be established.

In the intricate interplay between mental health and economic uncertainty, intolerance to uncertainty (IU) stands as a pivotal psychological factor. IU, the tendency to react negatively to ambiguous situations, is a significant contributor to mental disorders (Boswell et al., 2013). It is suggested that individuals with high IU exhibit a cognitive bias that interprets uncertainty as threatening, which may predispose them to anxiety disorders, major depressive disorder, and eating disorders (Brown et al., 2017; Rettie & Daniels, 2021; Sahib et al., 2023). The perception and cognitive interpretation of economic uncertainty, influenced by IU, could indeed amplify these disorders. In this context, Carleton's (2016) review highlights the transdiagnostic significance of fear of the unknown, a concept central to models of emotion, attachment, and neuroticism, emphasizing the universal impact of uncertainty on anxiety-related disorders. With the current global economic shifts, the role of IU in shaping psychological resilience or vulnerability is increasingly recognized. The present study's exploration into in the context of economic uncertainty thus draws a parallel to individual-level IU, offering insights into how economic conditions may act as a stressor, triggering or predisposing mental health issues.

This study uses the World Uncertainty Index (WUI) (Ahir et al., 2022) as a novel metric to examine the relationship between economic uncertainty and mental disorders globally. The WUI serves as an indirect yet insightful indicator of global economic sentiment (Er et al., 2023). The term ‘economic uncertainty,’ distinctively chosen over the more general ‘world uncertainty,’ reflects explicitly the unpredictability in economic outcomes from varied sources, such as political upheavals, natural disasters, financial crises, or significant policy shifts, central themes in the Economist Intelligence Unit (EIU)^1^ reports (Ahir et al., 2022). In the past three decades, as presented in Ahir et al.'s (2022) study, notable spikes in the WUI have aligned with significant incidents like the 9/11 attacks, Gulf War II, Brexit, the 2016 US presidential elections, and US-China trade tensions. Notably, the onset of the COVID-19 pandemic was a historical peak in 2020 due to uncertainty. Also, the WUI across countries shows that the level of uncertainty varies significantly, being generally lower in advanced economies (Ahir et al., 2022). Studies by Er et al. (2023) and Liu (2023)reinforce the understanding of economic uncertainty patterns, highlighting the WUI's relevance in public health.

In this study, we empirically estimate the relationship between economic uncertainty and three common mental disorders: anxiety disorders, major depressive disorder, and eating disorders, for the period of 1991–2019. We focus on these mental health conditions because they are common across the world, with significant burdens on public health and plausibly sensitive to economic uncertainty (Carleton, 2016). Anxiety disorders and major depressive disorders are, in fact, leading common mental health conditions in the world today (Global Burden of Disease Collaborative Network, 2020). Eating disorders are less frequent, but their gravity is no less, which makes a serious burden on health and have one of the highest risks of concomitant occurrence with other mental health morbidities (Barakat et al., 2023; Brown et al., 2017). Moreover, we explore whether such economic uncertainty affects mental health differently by gender and age groups. Finally, our study is comprehensive in understanding the detailed ways in which economic uncertainty results in an adverse effect on mental health, contributing to shaping targeted and effective policies for public health intervention.

## 2 Data

We used data on mental disorders from the Global Burden of Disease Study 2019 (GBD) (Global Burden of Disease Collaborative Network, 2020). It is a comprehensive study that presents data on several health conditions and risks for all countries, critically exploring all distinctions between global and regional health trends and challenges (Diaz et al., 2021; Murray, 2022; GBD 2019 also includes mental health-related disorders that we adopted, further divided into anxiety disorders, major depressive disorder, and eating disorders (Javaid et al., 2023; Santomauro et al., 2021). We used data approximated prevalence rates^2^ to reflect the total burden of mental disorders, chronic and new cases combined, within a given year, for these mental disorders per 100,000 populations from 1991 to 2019. So, the prevalence rate indicates the full scope of mental health disorders in the population, providing a comprehensive metric for public health planning and resource allocation (Santomauro et al., 2021). This approach aligns with epidemiological studies (GBD 2019 Mental Disorders Collaborators, 2022; Santomauro et al., 2021), which also use prevalence as a critical indicator for mental health surveillance and policy response in the face of economic uncertainty (Frasquilho et al., 2015).

In our analysis, we utilized the WUI as a key metric for economic uncertainty. The WUI stands out from similar indices due to its unified methodology and source consistency, enhancing its suitability for cross-national evaluations. The index is constructed on a quarterly basis, tracking the prevalence of the term ‘uncertainty’ within the comprehensive reports of EIU) (https://www.eiu.com/). Normalization against total word counts ensures that the WUI is equitable across nations. Ahir et al. (2022) affirm the index's robustness across various economic strata, notwithstanding the more prolonged reports commonly found in more affluent countries. To accommodate our annual analytical framework, we averaged the index's quarterly values, like Er et al. (2023).

Furthermore, we gathered control variables from the World Bank Databank (WBD) (World Bank, 2023). First, Gross Domestic Product (GDP) per capita (current US dollar ($)), adjusted for purchasing power parity and expressed on a natural logarithmic scale (ln), served as a proxy for income level, which is intricately linked to mental well-being (Er et al., 2023). Second, unemployment rates were included as a measure of economic stability, with higher rates often correlating with increased stress and mental health issues (de Bruin et al., 2020). Third, population size, on a natural logarithmic scale (ln), was factored in to normalize the data across countries of varying sizes and to account for the societal impacts of population dynamics on mental health (Er et al., 2023). In addition, our study's rigor was augmented by employing the principles outlined in the Guidelines for Accurate and Transparent Health Estimates Reporting (GATHER).

### 2.1 Study sample

**Fig. 1:**
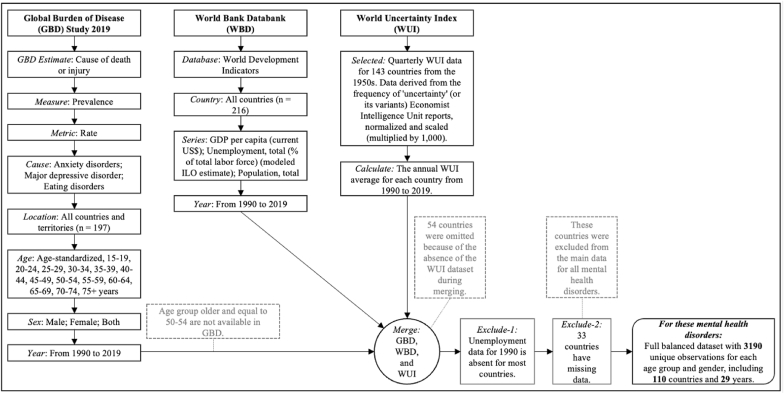
Data management flowchart.

In our study, detailed in Fig. 1, we used a comprehensive dataset from the 1991 to 2019 timeframe for 110 countries. By the GBD data, we utilized age-standardized prevalence rates for anxiety disorders, major depressive disorder, and eating disorders, segmented into specified age groups. The GBD Results tool provided the classifications, identifying anxiety disorders as code B.6.4 at level 3, major depressive disorder as B.6.2.1 at level 4, and eating disorders that cover anorexia nervosa and bulimia nervosa as B.6.5 at level 3 within the mental disorders category by sex, including female, male, and both of them (Global Burden of Disease Collaborative Network, 2020; Tao & Cheng, 2023; Tham et al., 2021; Xiong et al., 2022). The age groups consist of older adolescents (15–19 years), young adults (20–24 years), adults (25–59 years in 5-year increments), and older adults (60–75+ years in 5-year increments), in accordance with life-course health monitoring and policy development guidelines (Diaz et al., 2021; McGrath et al., 2023; Xiong et al., 2022).

To strengthen our study against potential biases, we handled the missing data by excluding 1990 due to the lack of unemployment data. Also, we removed 33 countries from our analysis because of their incomplete datasets. Additionally, we omitted 54 countries due to data mismatches between the GBD and the WUI during the data merging process. After these exclusions, our study encompassed a comprehensive dataset from 110 countries, ensuring a more robust and reliable analysis. As a result, we created a fully balanced dataset covering the years between 1991 and 2019 for this study. These measures were taken to minimize selection and information biases, leading to a consolidated dataset of 3190 unique observations per disorder, sex, and age group (including the age-standardized prevalence rate).

### 2.2 Summary statistics

We presented the summary statistics in Table 1. We found that the average age-standardized prevalence per 100,000 population for anxiety disorders stood at 4234 (SD = 1184), for the major depressive disorder at 2796 (SD = 910), and for eating disorders at 209 (SD = 150). The mean value for economic uncertainty was 2.45 (SD = 2.99). Our analysis also accounted for various economic and demographic measures as control variables, including the natural log of GDP per capita (average 8.03, SD 1.69), unemployment rates (average 7.18, SD 5.75), and log-transformed population figures (average 16.61, SD 1.36).

**Table 1:** Summary statistics and variable descriptions.

Variables	Mean/(SD) - N			min	max	Description	Data sources
	All	Female	Male				
**Dependent variables**							
Prevalence rate						Age-standardized mental disorders prevalence per 100,000 population.	Global Burden of Disease Collaborative Network (2020)
Anxiety disorders	4233.99	5284.16	3182.88	2001.30	8624.63	Anxiety disorders involve intense fear, distress, and physiological symptoms.	
	(1183.75)	(1682.29)	(756.03)				
Major depressive disorder	2796.13	3457.99	2121.33	707.95	6444.10	Major depressive disorder is a recurrent mood disorder marked by prolonged daily episodes of depressed mood or loss of interest, lasting at least two weeks.	
	(909.69)	(1106.10)	(781.43)				
Eating disorders	209.12	295.65	125.29	56.22	1031.69	Eating disorders, the aggregate measure of anorexia nervosa and bulimia nervosa, lead to both fatalities and disability due to abnormal eating behaviors and concerns about food, eating, and body image.	
	(150.19)	(232.93)	(77.13)				

**Variable of interest**							
Economic uncertainty	2.45			0.00	25.51	Economic uncertainty refers to the World Uncertainty Index (WUI), which is determined by the proportion of the word “uncertainty” or its variations found in country reports from the Economist Intelligence Unit.	Ahir et al. (2022)
	(2.99)						

**Control variables**						Country level control variables.	
Income	8.03			3.17	11.55	Income denotes the GDP per capita (in current US dollars) with a logarithmic transformation applied.	World Bank (2023)
	(1.69)						
Unemployment	7.18			0.09	37.98	Unemployment represents the proportion of joblessness within the total labor force, expressed as a percentage.	
	(5.75)						
Population	16.61			13.10	21.06	Population represents the natural logarithm of the total population.	
	(1.36)						
Year	29			1991	2019		
Countries	110						
Observations	3190						

*Notes*: The table presents summary statistics along with descriptions of variables. It presents the mean values (with standard deviations in parentheses), the number of observations (N), minimum and maximum values, descriptions of the variables, and the sources of data. The prevalence mean for each gender subsample is provided alongside all the samples. We presented the prevalence mean for each age group in Appendix Table A2.

**Fig. 2:**
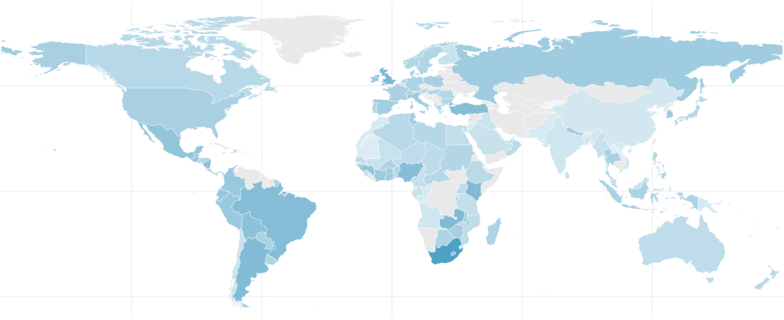
Average Economic Uncertainty by Country (1991–2019). *Notes:* This figure presents the average level of economic uncertainty per country over the period from 1991 to 2019 (for details, see Appendix Table A1), as indicated by the World Uncertainty Index (WUI). The variation in color intensity mirrors the average WUI values, with darker shades signifying greater uncertainty.

Fig. 2 presents a graphical illustration of the average level of economic uncertainty faced by countries around the world from 1991 to 2019, as measured by the WUI. The map employs varying shades of blue to reflect the WUI values, with darker tones representing higher levels of economic uncertainty. Countries such as those in South America, parts of Africa, and a few in the Middle East are shaded in the darkest blue, indicating a higher average economic uncertainty in these regions over the given period. In contrast, regions like North America, Australia, and some parts of Europe and Asia exhibit lighter shades, suggesting comparatively lower levels of economic uncertainty (for country details, see Appendix Table A1).

**Fig. 3:**
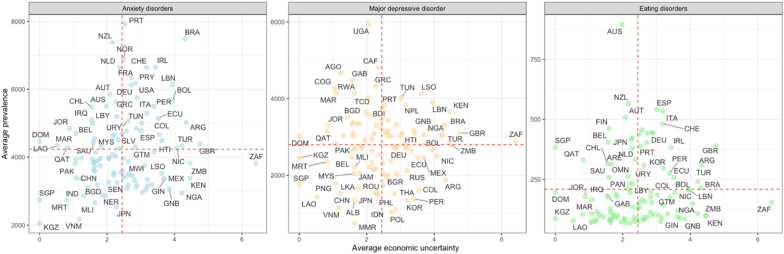
Global distribution of the age-standardized prevalence of anxiety disorders, major depressive disorder, and eating disorders and economic uncertainty (1991–2019). *Notes:* This figure presents a scatterplot analysis comparing the average prevalence of mental disorders with the average World Uncertainty Index (WUI) across countries from 1991 to 2019. It is structured into three panels, each representing a specific disorder: anxiety disorders, major depressive disorder, and eating disorders. The age-standardized prevalence of these disorders is plotted on the y-axis, and the economic uncertainty faced by each country is on the x-axis. The dashed lines in each panel indicate the global average prevalence and economic uncertainty for the respective disorders, serving as reference points to discern countries with above- or below-average values. Colors distinguish the disorders: blue for anxiety disorders, orange for major depressive disorder, and green for eating disorders.

Fig. 3 shows the distribution between the average prevalence of anxiety disorders, major depressive disorder, and eating disorders and the average economic uncertainty in countries from 1991 to 2019. Three scatterplots display the relationship, with the prevalence of disorders on the vertical axis and the economic uncertainty on the horizontal axis. Anxiety disorders, shown in blue, reveal high rates in Portugal (PRT) and New Zealand (NZL) against differing economic backgrounds, while Brazil (BRA) shows high anxiety prevalence with moderate economic uncertainty. South Africa (ZAF), despite its high economic uncertainty, has average anxiety levels. In the orange panel for major depressive disorder, Uganda (UGA) stands out with prevalence well above the global mean, hinting at complex socioeconomic dynamics beyond just economic uncertainty. The green panel on eating disorders shows lower overall rates, but Australia (AUS) has a notably higher prevalence, suggesting factors such as cultural norms and reporting practices play a role. The data collectively suggests that while economic uncertainty and mental health are linked, the connection is nuanced, with other social and healthcare factors significantly influencing mental health outcomes.

## 3 Methodology

We employed a fixed effects model with robust standard errors in our comprehensive analysis of the relationship between economic uncertainty and mental disorder prevalence (Kawachi et al., 2023; Liu, 2023; Sari et al., 2023). Equation (1) displays the adopted model:

(1) $${Mental\;Disorder}_{i,t}=a+\beta_{1}{WUI}_{i,t}+\beta_{2}X_{i,t}+z_{i}+y_{t}+\varepsilon_{i,t}$$

where ${Mental\;Disorder}_{i,t}$ is the prevalence rate per 100,000 populations of the mental disorders for country i at year t for anxiety disorders, major depressive disorders, and eating disorders, separately. ${WUI}_{i,t}$ denotes the World Uncertainty Index, economic uncertainty, with higher values indicating greater uncertainty. $X_{i,t}$ is the vector of control variables, including income, unemployment rate, and total population. $z_{i}$ is the country fixed effect, $y_{t}$ is the year-fixed effect, and $\varepsilon_{i,t}$ is the error term. In our statistical approach, multivariate adjustments within the fixed effects model ensure that unobserved country-specific characteristics that do not change over time and could potentially confound the relationship between economic uncertainty and mental health are controlled for. Thus, it allows us to isolate the specific relationship of economic uncertainty with mental health, thereby providing a more accurate estimation of the true outcomes and a clearer understanding of these relationships. Further enhancing our analysis, all models were estimated using robust standard errors to correct for heteroscedasticity and autocorrelation within panels. Besides, our analysis indicates no significant multicollinearity among the variables in the models, as evidenced by the eigenvalues derived from the correlation matrix.

Initially, we assessed the immediate effect of economic uncertainty on the prevalence rates of mental disorders per 100,000 population. Subsequently, we conducted a sensitivity analysis by examining the 1-year lagged relation of the WUI_(t-1)_ on the prevalence of mental disorders to assess the robustness of our findings to potential delayed results of economic uncertainty. This analysis allowed us to determine whether the associations observed were consistent over time, providing insight into the temporal dynamics of economic uncertainty on mental health. As a further analysis, we employed subgroup analysis by gender by using the same control variables and year-fixed effect. In the final analytical phase, we extended our analyses to dissect these age-specific patterns across all age groups, separately employing the same model: older adolescents, young adults, adults, and older adults.

## 4 Findings

In this analysis, the primary focus is on the prevalence rates of anxiety disorders, major depressive disorders, and eating disorders as they relate to fluctuations in the economic uncertainty, WUI, across 110 countries between 1991 and 2019. The estimates are presented in Table 2, and we report the associations with a one standard deviation increase in the WUI to offer a clearer understanding of how variations in the WUI relate to changes in mental health outcomes.

**Table 2:** The association between anxiety disorders, major depressive disorder, and eating disorders: prevalence rates and economic uncertainty.

Variables	*Dependent variables (Age-standardized prevalence* per *100,000 population)*					
	Anxiety disorders		Major depressive disorder		Eating disorders	
	(1)	(2)	(3)	(4)	(5)	(6)
WUI	3.602**		3.712*		−0.077	
	(1.590)		(2.065)		(0.195)	
WUI_(t-1)_		1.921		4.297**		−0.139
		(1.638)		(2.046)		(0.187)
Income	−11.838	−13.267	−28.867	−28.042	3.985*	3.919*
	(22.707)	(22.889)	(25.749)	(25.808)	(2.315)	(2.312)
Unemployment	10.969***	10.902***	−1.921	−1.984	−0.119	−0.118
	(3.841)	(3.848)	(4.411)	(4.387)	(0.369)	(0.368)
Population	−40.275	−42.147	−31.814	−30.896	−38.886***	−38.961***
	(50.423)	(50.917)	(87.187)	(87.321)	(11.118)	(11.115)

Country FE	YES	YES	YES	YES	YES	YES
Year FE	YES	YES	YES	YES	YES	YES
# Countries	110	109	110	109	110	109
# Observations	3190	3189	3190	3189	3190	3189
R^2^	0.027	0.025	0.008	0.009	0–088	0.089
F Statistic	20.842	19.332	5.779	6.563	73.962	74.131

*Notes*: The fixed-effects model coefficients demonstrate the relationship between economic uncertainty and the age-standardized prevalence of anxiety disorders, major depressive disorder, and eating disorders per 100,000 population across 110 countries. The primary measure of economic uncertainty is the World Uncertainty Index (WUI), analyzed both in its present-year form (Columns (1), (3), and (5)) and as a one-year lagged variable (WUI_(t-1)_) (Columns (2), (4), and (6)). Control variables include log-transformed GDP per capita (Income), the unemployment rate (Unemployment), and log-transformed total population (Population). Country-fixed effects (Country FE) and year-fixed effects (Year FE) are accounted for in all models. The reported R-squared (R^2^) values correspond to the within R-squared for our fixed effects panel data models. The table presents robust standard errors under parenthesis, which were calculated utilizing the Arellano robust variance estimator with country-level clustering to mitigate within-group error correlation. *p < 0.1; **p < 0.05; ***p < 0.01.

In Table 2, column (1) shows that a one standard deviation increase in the WUI,^3^ equivalent to 2.99 units, is associated with a significant increase of approximately 10.77 cases per 100,000 population in the prevalence of anxiety disorders. This result suggests that higher economic uncertainty is associated strongly with increased anxiety across various countries. Additionally, column (2) explores the delayed effects of economic uncertainty by analyzing the lagged WUI (WUI_(t-1)_). Here, the results indicate no significant association, which may imply that the relationship between economic uncertainty and anxiety disorders is more observable in the immediate term rather than in delayed effects. For major depressive disorders, as shown in column (3) of Table 2, a one standard deviation increase in the WUI correlates with a significant increase of approximately 11.09 cases per 100,000 in the prevalence rate. This finding underscores the relationship between economic uncertainty and increasing depressive symptoms. Furthermore, the lagged effect of the WUI, detailed in column (4), remains significant, with an increase of about 12.81 cases per 100,000 population, indicating a persistent and significant impact of economic uncertainty on major depressive disorder over time. In contrast, neither WUI (column (5)) nor lagged WUI (column (6)) shows a statistically significant association with the prevalence of eating disorders. In column (5), the coefficient of −0.077 per unit increase in WUI suggests a minimal and statistically insignificant relationship, even after scaling for one standard deviation. These results underscore the possibility that economic uncertainty may not have a direct or measurable relationship with the prevalence of eating disorders as it does with anxiety and depressive disorders.

In Table 2, the associations observed between the control variables—Income, Unemployment, and Population—and mental health outcomes vary. Higher unemployment rates are associated with increased reports of anxiety, reflecting the potential stress linked to joblessness. Similarly, higher GDP per capita (Income) tends to align with higher reported prevalence rates for eating disorders, suggesting a relationship between GDP per capita and eating disorders. The relationship with Population indicates that larger population sizes are correlated with lower reported prevalence rates of eating disorders, which could be related to factors like more comprehensive healthcare resources. These associations underscore the importance of considering economic and demographic factors in the analysis of mental health across different countries. Additionally, we followed Ruhm (2000, 2015) and included the country-specific linear time trends in our analyses.^4^ These new results, which we present in Appendix Table 3A, did not alter the significant associations observed between economic uncertainty and anxiety and major depressive disorder outcomes.

For the analysis focused on females, as presented in Table 3 (for details, Appendix Table 4A), the results indicate a distinct pattern of association between economic uncertainty and the prevalence of mental health disorders. Column (1) of the table shows that a one standard deviation increase in the WUI is associated with an increase in the prevalence of anxiety disorders among females by approximately 11.32 cases per 100,000 population. This result shows that in the context of increasing economic uncertainty, anxiety levels among females tend to rise. In columns (2) and (3), the results show that there is no significant association between economic uncertainty and the prevalence of major depressive disorder and eating disorders among females. Once we focused on males, in Table 4, we see a similar pattern with females in anxiety disorders.

**Table 3:** The association between economic uncertainty and anxiety disorders, major depressive disorder, and eating disorders prevalence rates among females.

Variables	*Dependent variables (Age-standardized prevalence* per *100,000 population)*		
	Anxiety disorders	Major depressive disorder	Eating disorders
	(1)	(2)	(3)
WUI	3.785*	4.270	−0.069
	(1.997)	(2.780)	(0.281)

Control variables	YES	YES	YES
Country FE	YES	YES	YES
Year FE	YES	YES	YES
# Countries	110	110	110
# Observations	3190	3190	3190
R^2^	0.021	0.007	0.058
F Statistic	16.045***	5.751***	50.847***

*Notes*: This table presents the estimated relationship between economic uncertainty and the age-standardized prevalence of anxiety disorders, major depressive disorder, and eating disorders, specifically analyzing data corresponding to females. The primary variable of interest, economic uncertainty, is measured by the World Uncertainty Index (WUI). Country-level clustered robust standard errors are presented under parenthesis. Country-fixed effects (Country FE) and year-fixed effects (Year FE) are accounted for in all models. The reported R-squared (R^2^) values correspond to the within R-squared. For more details, see Appendix Table A4. *p < 0.1; **p < 0.05; ***p < 0.01.

**Table 4:** The association between economic uncertainty and anxiety disorders, major depressive disorder, and eating disorders prevalence rates among males.

Variables	*Dependent variables (Age-standardized prevalence* per *100,000 population)*		
	Anxiety disorders	Major depressive disorder	Eating disorders
	(1)	(2)	(3)
WUI	2.774**	2.707*	−0.112
	(1.229)	(1.543)	(0.120)
			
Control variables	YES	YES	YES
Country FE	YES	YES	YES
Year FE	YES	YES	YES
# Countries	110	110	110
# Observations	3190	3190	3190
R^2^	0.024	0.008	0.058
F Statistic	18.462***	5.979***	47.242***

*Notes*: This table presents the estimated relationship between economic uncertainty and the age-standardized prevalence of anxiety disorders, major depressive disorder, and eating disorders, specifically analyzing data corresponding to males. The primary variable of interest, economic uncertainty, is measured by the World Uncertainty Index (WUI). Cluster-robust standard errors are presented in parentheses, with country-level clustering. Country fixed effects (Country FE) are included in all models, and year-fixed effects (Year FE) are added in Columns (2), (4), and (6). The reported R-squared (R^2^) values correspond to the within R-squared. For more details, see Appendix Table A5. *p < 0.1; **p < 0.05; ***p < 0.01.

Table 4 indicates a positive correlation between economic uncertainty, anxiety disorders, and major depressive disorder in males. The estimated associations reveal that a one standard deviation increase in the WUI leads to an increase of approximately 8.28 cases for anxiety disorders (column (1)) and about 8.07 cases per 100,000 for major depressive disorders (column (2)). This pattern is slightly less pronounced than that observed among females for anxiety disorders. For eating disorders (column (3)), the WUI does not appear to have a significant association among males, which aligns with the findings for females. For more information, please refer to Appendix Table A5.

**Fig. 4:**
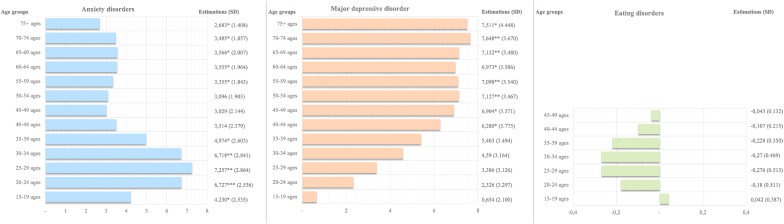
The association between economic uncertainty and age-specific anxiety disorders, major depressive disorder, and eating disorders prevalence. *Note:* This figure shows the relationship between economic uncertainty and the prevalence of three mental disorders – anxiety, major depressive disorder, and eating disorders – across various age cohorts. The bar charts represent the fixed-effects model estimates of prevalence per 100,000 population in a country, with error bars indicating 95% confidence intervals, thus reflecting the precision of the estimates. This multifaceted analysis includes control variables for income, unemployment rates, population sizes, and year-fixed effects to account for confounding factors. The eating disorders' scope is confined to ages 15–49 due to data limitations in GBD 2019 and, due to DSM diagnosis criteria, the diminished incidence of eating disorders post-49 aligning with established epidemiological patterns. The visual presentation underscores the critical role of economic factors in correlation with mental health outcomes across the lifespan. The prevalence estimates are displayed alongside their statistical significance, denoted by asterisks, where *** indicates p < 0.01, ** denotes p < 0.05, and * indicates p < 0.1. For more details, see Appendix Tables A6, A7, and A8.

Acknowledging the complexities of economic uncertainty on mental health across various age cohorts, Fig. 4 offers an analytical presentation of the associations between the prevalence of anxiety disorders, major depressive disorders, and eating disorders and the WUI. The bar charts frame the estimations of fixed-effects models, the same as the main model but specific for each age cohort. The age-specific examination, underpinned by robust standard errors and inclusive of country and year-fixed effects, elucidates nuanced relationships that vary by age and mental health condition. In terms of anxiety disorders, represented in blue, the analysis indicates a noticeable association across several age groups. For instance, prevalence estimates show significance in younger cohorts (from 15 to 19 to 35 to 39) and consistently from 55 to 59 onwards. This trend may reflect varying responses to economic uncertainty across the lifespan, where early and later life stages show a noticeable correlation with uncertainty, suggesting possible periods of increased vulnerability. The orange bars corresponding to major depressive disorder exhibit a persistent pattern of association with WUI across a broad age spectrum. In particular, middle-aged and older cohorts (50–54 and onwards) display prevalence estimates with statistical significance, potentially underscoring the cumulative or heightened sensitivity to economic uncertainty factors with advancing age. Regarding eating disorders, constrained by data to ages 15–49 and depicted in green, the association with economic uncertainty is less evident within the analyzed age groups, as indicated by the lack of statistically significant increments in prevalence estimates. See Appendix Tables A6, A7, and A8 for the detailed results.

## 5 Discussion

In this global analysis, we showed the insights from the World Uncertainty Index (WUI) to evaluate the prevalence of a trio of mental health conditions—namely anxiety disorders, major depressive disorders, and eating disorders—over a span encompassing nearly three decades and 110 countries. Our main findings show a significant association between economic uncertainty and mental health across specific age cohorts. Notably, anxiety disorders show a pronounced correlation with economic uncertainty in older adolescents, young adults, and adults in early ages, including ages 35–39, and then older adults within the 55–59 age bracket. We see no significant association for the adult ages covering the 40–54 age brackets.^5^ Besides, major depressive disorder demonstrates significant associations in age groups 50–54 and older. Conversely, we found the relationship between economic uncertainty and the prevalence of eating disorders across none of the age groups is significant. This nuanced understanding highlights how economic uncertainty may differentially affect mental health depending on the stage of life, suggesting age-specific vulnerabilities and resilience patterns in the face of economic uncertainty.

In studying the complex relationship between economic uncertainty and anxiety disorders, major depressive disorders, and eating disorders, our research revealed distinct patterns of association across different demographics. The use of the WUI showed a clear relation between anxiety disorders, major depressive disorders, and economic uncertainty, with notable variations by gender and age. This finding affirms a deeper exploration into the socio-economic and cultural factors that may amplify the risk of mental disorders among women in uncertain economies (Javaid et al., 2023; Tao & Cheng, 2023). Conversely, for females, the study identified a significant association solely with anxiety disorders. This raises questions about the gender-specific mechanisms through which uncertainties in the economic climate may be associated with mental well-being and the role of societal expectations and support systems in mediating these effects (Tham et al., 2021). Besides, as highlighted by Xiong et al. (2022), females may be influenced by hormonal fluctuations experienced during various reproductive stages, such as puberty, menstruation, pregnancy, postpartum, and menopause, which have been shown to make women particularly vulnerable to these conditions. Abdoli et al. (2022) also underline the effect of biopsychosocial factors leading to a higher susceptibility of females to major depressive disorder than males, but we see no significant relationship between economic uncertainty and major depressive disorders among females. This disparity might be attributed to the greater financial and social responsibilities often assumed by the elderly, particularly men over 45, who may experience heightened stress from economic uncertainties due to nearing retirement or increased family obligations (Silva et al., 2020).

Our age-stratified analysis for anxiety disorders provides pivotal insights in line with existing literature that identifies the prevalence among the age groups (Xiong et al., 2022). Also, our finding resonates with prior research indicating that economic fluctuations can profoundly affect individuals in their prime working years when they are likely to establish careers (Tao & Cheng, 2023), and possibly start families—activities that economic uncertainty can severely disrupt (Ridley et al., 2020). For major depressive disorder, the observed increase in prevalence among individuals aged 50 and over corroborates with the growing body of evidence that suggests late-life depression is often a product of complex interactions between social, economic, and health-related factors (Abdoli et al., 2022). The accumulative effect of economic uncertainty over a lifetime may lead to an increased burden of major depressive disorder, compounded by the challenges of aging, such as social isolation, loss of independence, and the prevalence of chronic health conditions (Abdoli et al., 2022). The vulnerability of young adults to eating disorders is also reflected in the literature (Brown et al., 2017), which uncertainty stressors as worsening factors for mental health issues that typically emerge during adolescence and young adulthood. Although our study finds no significant relationship between economic uncertainty and eating disorders, the association between higher GDP per capita and the prevalence of eating disorders implies that such conditions may be more prevalent in wealthier countries, suggesting an indirect association where economic factors can still influence the reporting of eating disorders. Overall, as noted by Barakat et al. (2023), these conditions are complex and are influenced by a wide range of environmental and psychological factors. These age-specific associations draw attention to the critical need for economic and mental health policies to be tailored to the life course.

Building on our extensive analysis, we recognize that controlling for GDP per capita and unemployment rates captures direct economic conditions. Nevertheless, WUI provides a broader perspective on economic uncertainty that goes beyond these metrics. This index reflects global economic sentiment by measuring the frequency of discussions on uncertainty within the Economist Intelligence Unit reports, encompassing not just financial indicators but also political, social, and international factors that might affect the collective economic outlook (Ahir et al., 2022). Moreover, the WUI's broader economic implications could contribute to heightened collective anxiety, reflecting a pervasive fear of future uncertainties not immediately apparent through GDP or unemployment data. This ties into the psychological concept of Intolerance of Uncertainty, which suggests that a high societal perception of uncertainty might disproportionately influence anxiety levels, thereby explaining the observed significant associations with anxiety disorders in our findings (Carleton, 2016). It reflects how perceptions of instability, related to economic indicators, can amplify anxiety symptoms, showing the complex interplay between macroeconomic conditions and individual psychological resilience. These insights highlight the complex interplay between economic conditions and mental health, suggesting that perceived economic instability could have distinct impacts on mental health outcomes, particularly anxiety, which is closely linked to fear and apprehension about the future. Thus, while traditional economic indicators remain important, our study underscores the importance of considering broader measures like the WUI to fully understand the implications of economic uncertainty on mental health. The results of our study highlight the necessity for mental health professionals to consider global and country-specific economic uncertainty when addressing mental health conditions. This reflective approach can help tailor interventions sensitive to the economic context of the individuals and communities they serve.

The extensive temporal and geographical scope of the data analyzed bolsters the generalizability of our findings. These findings offer important considerations for global health policy, suggesting a valuable direction for public health initiatives to address the association between economic uncertainty and mental health on a worldwide scale. Also, as Herrman et al. (2022) pointed out, including mental health in universal health coverage plans and recognizing the need for cross-sectoral policies and interventions is important to reduce the adverse effect of uncertainty. It is important for policymakers to recognize that the economic environment is strongly linked to the population's mental health (Er et al., 2023; Liu, 2023; Tham et al., 2021). The associations we observed call for a multidisciplinary approach that integrates economic planning with mental health strategies. For health policymakers, the evidence underscores the need to safeguard mental health during economically uncertain times through supportive policies, such as strengthening social safety nets, ensuring access to mental health services, and investing in community-based initiatives to provide social support. However, while our study provides valuable global insights, health policymakers must also consider regional nuances in economic conditions, healthcare infrastructure, and cultural attitudes toward mental health.

Our study benefits from several significant strengths. Firstly, the large-scale, multicountry dataset from the Global Burden of Disease Study offers a comprehensive overview of mental disorders across diverse economic contexts. This extensive coverage enhances the generalizability of our findings to a global population. Additionally, using the WUI as a measure of economic uncertainty is a novel approach that provides a standardized, internationally comparable metric. While our study leverages the WUI, which quantifies economic uncertainty through the analysis of terms in structured EIU reports, it also highlights a broader opportunity for the media. Careful and factual media coverage on economic issues can play a complementary role, helping to shape public perceptions responsibly and mitigate anxiety related to economic uncertainty. Furthermore, the utilization of fixed effects regression in our analysis is a pivotal strength, as it allows us to mitigate confounding from stable, unmeasured differences by comparing countries to themselves over time, thereby reinforcing the robustness of our findings.

While the study's extensive dataset and robust analytical approach lend credence to these observations, limitations need caution in interpretation. We acknowledge the risk of ecological fallacy inherent in aggregate-level analyses (Sari et al., 2023). Furthermore, we recognize the constraints that come with relying on the WUI to measure economic uncertainty and the GBD data for mental health estimates across countries. Although these instruments have standardized formats, they may only partially capture the complex aspects of economic uncertainty and the variances in health diagnostics, which can impact the reliability of cross-country comparisons. Our study's reliance on such data necessitates cautious interpretation, especially when comparing countries with vast differences in economic situations and healthcare systems. To mitigate this risk, we incorporated stratification by age and sex, allowing us to examine more nuanced patterns and relationships that might be observed in a broader analysis. Although our analysis focuses on the period from 1991 to 2019, preliminary exploratory analyses for shorter periods (2005–2019 and 2010–2019) suggest potential variability in the relationship between economic uncertainty and mental health outcomes. These results were influenced by a significant reduction in sample size, limiting our ability to detect stable effects.

Additionally, using the WUI enabled us to explore these relationships within a consistent framework across various countries and economic contexts. Although the relationship between economic uncertainty and mental disorders is not uniform across individuals, as indicated Sari et al. (2023), the significance of aggregate-level analysis in elucidating variations in large-unit data cannot be overlooked. Also, like Er et al. (2023), we acknowledge that the potential for bias arising from variability in reporting standards and diagnostic criteria for mental disorders across countries and years cannot be discounted. Additionally, binge eating disorder, recognized as the predominant form of eating disorder, is linked with substantial health consequences (Bottera et al., 2024). However, due to its absence in the GBD study, we were unable to incorporate it into our analysis.

## 6 Conclusion

Our research contributes to the global understanding of the nexus between economic uncertainty and mental health. By transcending the limitations of localized or clinical studies and incorporating stratification by gender and age, our study highlights the widespread effects of economic uncertainties on mental health. It thereby empirically reinforces theories that position uncertainty as a catalyst for adverse health outcomes, including mental disorders. Our findings signal the importance of integrating uncertainty into mental health research and tailoring public health interventions and prevention to different life courses. To further enrich our understanding, future studies could explore the role of cultural dimensions, such as Hofstede's Uncertainty Avoidance Index, in moderating the effects of economic uncertainty on mental health. This could provide nuanced insights into how cultural predispositions towards uncertainty influence psychological resilience or vulnerability across different societies. Future research should continue to explore these relationships, with a focus on refining the gender-specific and age-related implications of other types of uncertainties on mental health that are culturally sensitive and demographically specific.

## Funding

This research project was funded by UiT the Arctic University of Norway, Norway

## Ethical statement

This study involves secondary data analysis and did not require the ethical review board approval, as it did not involve direct data collection from human or animal subjects. The data obtained from publicly available sources were anonymized and collected by ethical standards. No identifiable personal information was accessed. There are no conflicts of interest to declare.

## CRediT authorship contribution statement

**Emre Sarı:** Writing – review & editing, Writing – original draft, Visualization, Software, Project administration, Methodology, Formal analysis, Data curation, Conceptualization, Supervision. **Buse Şencan Karakuş:** Writing – review & editing, Writing – original draft, Data curation, Conceptualization. **Ender Demir:** Writing – review & editing, Writing – original draft, Methodology, Conceptualization.

## Declaration of generative AI and AI-assisted technologies in the writing process

During the preparation of this work, the author(s) used Grammarly and OpenAI in order to improve the readability and language of the manuscript. After using this tool/service, the author(s) reviewed and edited the content as needed and take(s) full responsibility for the content of the publication.

## Declaration of competing interest

We declare no competing interests.

## Data Availability

All datasets relevant to this study are publicly available. Global Burden of Disease Study 2019 data are available from https://vizhub.healthdata.org/gbd-results/, the World Uncertainty Index data from https://worlduncertaintyindex.com/, and the World Bank Databank data from https://databank.worldbank.org/.

## Footnotes

1 The Economist Intelligence Unit is a prestigious worldwide research and analysis department of The Economist Group (https://www.eiu.com/). For nearly eight decades, they have provided valuable insights into economic indicators, country analyses, and global trends. This formulation ensures that the time trend for each country starts at 1 and increases by 1 for each subsequent year, capturing linear temporal effects within each country.

2 The prevalence rates for mental disorders from the Global Burden of Disease Study 2019 are derived using a complex methodology that includes epidemiological data, meta-analyses, and proprietary models to adjust for different diagnostic criteria and reporting standards across countries. These efforts aim to standardize estimates, though variations in healthcare quality, cultural perceptions, and diagnostic practices may affect the accuracy of cross-country comparisons. For detailed methodological descriptions and the associated challenges, please refer to Ferrari et al. (2024), Murray (2022) and Vos et al. (2020), and their respective appendices.

3 Estimation of the association from one standard deviation increase in WUI: The association between a one standard deviation increase in the WUI and the prevalence of each mental disorder is estimated by multiplying the regression coefficient of the WUI by its standard deviation. This is represented mathematically as: $\Delta Prevalence=\beta \times \sigma_{WUI}=3.602\times 2.99$ where β is the coefficient of the WUI from the regression model, and $\sigma_{WUI}$ is the standard deviation of the WUI.

4 We introduce country-specific linear time trends into our fixed-effects model as a sensitivity check to account for potential unobserved heterogeneity that may vary across countries and over time within each country. Mathematically, this is represented by adding a time trend variable for each country, defined as: $T_{i,t}=t-t_{i,1}+1.\;T_{i,t}$, represents the time trend for country i at time t, and $t_{i,1}$ is the starting year for country i in the dataset.

5 The definitions for age groups have been determined by following Diaz et al. (2021).
