# Supplementary material for: Economic uncertainty and mental health: Global evidence, 1991 to 2019
Source: SSM Popul Health. 2024 Jun 14;27:101691. doi: 10.1016/j.ssmph.2024.101691 (PMC11231563; doi:10.1016/j.ssmph.2024.101691)
Supplement: Multimedia component 1 [file mmc1.docx]

Online Appendix

**Economic Uncertainty and Mental Health:**

*Global evidence, 1991 to 2019.*

*by*

Emre Sarı, Buse Şencan Karakuş, and Ender Demir^*^

*Oslo*

June 2024

^*^*Correspondence to:* Division for Health and Social Sciences, NORCE Norwegian Research Centre, Tullins gate 2, 0166 Oslo, Norway. [emre@norceresearch.no](mailto:emre@norceresearch.no)

**Table A1** – Average economic uncertainty (WUI) at the country level between 1991 and 2019.

| **Country name** | **ISO codes** | **Average WUI** | **Country name** | **ISO codes** | **Average WUI** | **Country name** | **ISO codes** | **Average WUI** |
| --- | --- | --- | --- | --- | --- | --- | --- | --- |
| Albania | ALB | 1.633 | Jamaica | JAM | 1.801 | Switzerland | CHE | 3.164 |
| Algeria | DZA | 2.223 | Japan | JPN | 2.280 | Thailand | THA | 2.816 |
| Angola | AGO | 1.232 | Jordan | JOR | 0.887 | Togo | TGO | 2.417 |
| Argentina | ARG | 4.340 | Kenya | KEN | 4.462 | Tunisia | TUN | 3.023 |
| Australia | AUS | 1.980 | Kyrgyzstan | KGZ | 0.000 | Türkiye | TUR | 4.226 |
| Austria | AUT | 2.085 | Lao People's Democratic Republic | LAO | 0.528 | Uganda | UGA | 2.055 |
| Bangladesh | BGD | 1.423 | Lebanon | LBN | 3.945 | United Arab Emirates | ARE | 1.661 |
| Belgium | BEL | 1.564 | Lesotho | LSO | 3.624 | United Kingdom | GBR | 4.752 |
| Benin | BEN | 2.630 | Libya | LBY | 2.288 | United Republic of Tanzania | TZA | 1.793 |
| Bolivia (Plurinational State of) | BOL | 3.943 | Madagascar | MDG | 2.632 | United States of America | USA | 2.888 |
| Botswana | BWA | 2.654 | Malawi | MWI | 3.123 | Uruguay | URY | 2.621 |
| Brazil | BRA | 4.314 | Malaysia | MYS | 1.632 | Viet Nam | VNM | 1.179 |
| Bulgaria | BGR | 2.490 | Mali | MLI | 1.645 | Zambia | ZMB | 4.458 |
| Burkina Faso | BFA | 2.593 | Mauritania | MRT | 0.813 | Zimbabwe | ZWE | 2.887 |
| Burundi | BDI | 2.453 | Mexico | MEX | 3.691 |  |  |  |
| Côte d'Ivoire | CIV | 3.322 | Morocco | MAR | 1.085 |  |  |  |
| Cameroon | CMR | 1.713 | Mozambique | MOZ | 2.058 |  |  |  |
| Canada | CAN | 2.259 | Myanmar | MMR | 1.641 |  |  |  |
| Central African Republic | CAF | 2.336 | Nepal | NPL | 3.152 |  |  |  |
| Chad | TCD | 2.049 | Netherlands | NLD | 2.368 |  |  |  |
| Chile | CHL | 1.553 | New Zealand | NZL | 2.164 |  |  |  |
| China | CHN | 1.228 | Nicaragua | NIC | 4.080 |  |  |  |
| Colombia | COL | 3.544 | Niger | NER | 1.943 |  |  |  |
| Costa Rica | CRI | 2.156 | Nigeria | NGA | 4.220 |  |  |  |
| Democratic Republic of the Congo | COG | 1.056 | Norway | NOR | 2.485 |  |  |  |
| Denmark | DNK | 2.876 | Oman | OMN | 2.158 |  |  |  |
| Dominican Republic | DOM | 0.000 | Pakistan | PAK | 1.083 |  |  |  |
| Ecuador | ECU | 3.512 | Panama | PAN | 2.200 |  |  |  |
| Egypt | EGY | 1.827 | Papua New Guinea | PNG | 1.083 |  |  |  |
| El Salvador | SLV | 2.832 | Paraguay | PRY | 2.838 |  |  |  |
| Ethiopia | ETH | 2.194 | Peru | PER | 3.383 |  |  |  |
| Finland | FIN | 1.591 | Philippines | PHL | 2.331 |  |  |  |
| France | FRA | 2.725 | Poland | POL | 2.862 |  |  |  |
| Gabon | GAB | 1.573 | Portugal | PRT | 2.525 |  |  |  |
| Germany | DEU | 2.717 | Qatar | QAT | 0.829 |  |  |  |
| Ghana | GHA | 3.051 | Republic of Korea | KOR | 3.195 |  |  |  |
| Greece | GRC | 2.267 | Romania | ROU | 2.292 |  |  |  |
| Guatemala | GTM | 3.396 | Russian Federation | RUS | 3.193 |  |  |  |
| Guinea | GIN | 3.692 | Rwanda | RWA | 1.641 |  |  |  |
| Guinea-Bissau | GNB | 3.840 | Saudi Arabia | SAU | 1.590 |  |  |  |
| Haiti | HTI | 3.624 | Senegal | SEN | 1.855 |  |  |  |
| Honduras | HND | 2.475 | Sierra Leone | SLE | 1.603 |  |  |  |
| Hungary | HUN | 2.579 | Singapore | SGP | 0.000 |  |  |  |
| India | IND | 1.311 | South Africa | ZAF | 6.412 |  |  |  |
| Indonesia | IDN | 2.734 | Spain | ESP | 3.006 |  |  |  |
| Iraq | IRQ | 1.542 | Sri Lanka | LKA | 1.733 |  |  |  |
| Ireland | IRL | 3.430 | Sudan | SDN | 2.447 |  |  |  |
| Italy | ITA | 3.177 | Sweden | SWE | 2.574 |  |  |  |

**Table A2** – Summary statistics for age groups.

| Age groups | Anxiety disorders | | Major depressive disorder | | Eating disorders | |
| --- | --- | --- | --- | --- | --- | --- |
|  | Mean | SD | Mean | SD | Mean | SD |
| 15-19 | 5361.3 | 1744.4 | 2496.85 | 896.41 | 402.5 | 281.52 |
| 20-24 | 5449.33 | 1600.58 | 3367.25 | 1165.02 | 532.38 | 387.72 |
| 25-29 | 5407.17 | 1575.63 | 3292.53 | 1135.49 | 523.15 | 383.38 |
| 30-34 | 5364.74 | 1576 | 3336.7 | 1174.39 | 486.03 | 341.2 |
| 35-39 | 5325.95 | 1574.98 | 3648.39 | 1284.44 | 378.56 | 264.97 |
| 40-44 | 5283.29 | 1569.34 | 3917.55 | 1373.13 | 224.92 | 175.8 |
| 45-49 | 5190.68 | 1523.34 | 4108.49 | 1426.84 | 123.73 | 113.43 |
| 50-54 | 5063.96 | 1451.72 | 4296.77 | 1524.68 |  |  |
| 55-59 | 4925.73 | 1382.57 | 4485.75 | 1658.8 |  |  |
| 60-64 | 4788.95 | 1332.56 | 4647.79 | 1802.16 |  |  |
| 65-69 | 4640.23 | 1287.68 | 4764.09 | 1956.25 |  |  |
| 70-74 | 4467.33 | 1233.14 | 4879.04 | 2152.08 |  |  |
| 75+ | 4055.61 | 1079.76 | 5101.48 | 2407.69 |  |  |
| *Note*: The table presents the mean values with standard deviations. | | | | | | |

**Table A3** - The association between anxiety disorders, major depressive disorder, and eating disorders: prevalence rates and economic uncertainty, including country-specific time trends.

|  | *Dependent variables (Age-standardized prevalence per 100,000 persons)* | | |
| --- | --- | --- | --- |
|  | Anxiety disorders | Major depressive disorder | Eating disorders |
| Variables | (1) | (2) | (3) |
| WUI | 3.582** | 3.706* | -0.071 |
|  | (1.602) | (2.090) | (0.194) |
| Income | -11.445 | -29.237 | 3.981* |
|  | (22.598) | (25.761) | (2.324) |
| Unemployement | 10.900*** | -1.831 | -0.120 |
|  | (3.873) | (4.403) | (0.369) |
| Population | -49.541 | -23.552 | -38.459*** |
|  | (47.904) | (86.138) | (11.146) |
| Country-specific time trend | 0.764 | -0.700 | -0.033 |
|  | (0.550) | (0.655) | (0.049) |
|  |  |  |  |
| Country FE | YES | YES | YES |
| Year FE | YES | YES | YES |
| # Countries | 110 | 110 | 110 |
| # Observations | 3190 | 3190 | 3190 |
| R^2^ | 0.028 | 0.009 | 0.089 |
| F Statistic | 17.724*** | 5.474*** | 59.381*** |
| *Notes*: The table presents results for the fixed-effects models, including country-specific time trends. The coefficients demonstrate the relationship between economic uncertainty and the age-standardized prevalence of anxiety disorders, major depressive disorder, and eating disorders per 100,000 population across 110 countries. Country-fixed effects (Country FE) and year-fixed effects (Year FE) are accounted for in all models. The reported R-squared (R^2^) values correspond to the within R-squared for our fixed effects panel data models. The table presents robust standard errors under parenthesis, calculated using the Arellano robust variance estimator with country-level clustering to mitigate within-group error correlation. *p<0.1; **p<0.05; ***p<0.01 | | | |

**Table A4** - The association between economic uncertainty and anxiety disorders, major depressive disorder, and eating disorders prevalence rates among females in detail.

|  | Dependent variables (Prevalence per 100,000 persons in a country) | | | | | |
| --- | --- | --- | --- | --- | --- | --- |
|  | Anxiety disorders | | Major depressive disorder | | Eating disorders | |
| Variables | (1) | (2) | (3) | (4) | (5) | (6) |
| WUI | 7.103*** | 3.785* | 1.684 | 4.270 | 1.545*** | -0.069 |
|  | (2.422) | (1.997) | (3.244) | (2.780) | (0.389) | (0.281) |
| Income | 39.248 | -12.121 | -93.321*** | -42.100 | 23.231*** | 3.951 |
|  | (23.899) | (31.248) | (24.410) | (34.365) | (3.756) | (3.630) |
| Unemployement | 13.932*** | 14.521*** | -2.907 | -2.954 | -0.469 | -0.262 |
|  | (5.270) | (5.365) | (6.450) | (6.499) | (0.525) | (0.526) |
| Population | 121.768*** | 23.503 | -123.593 | -56.312 | -3.510 | -47.310** |
|  | (40.118) | (66.214) | (75.353) | (118.845) | (11.524) | (21.469) |
|  |  |  |  |  |  |  |
| Country FE | YES | YES | YES | YES | YES | YES |
| Year FE | NO | YES | NO | YES | NO | YES |
| # of Countries | 110 | 110 | 110 | 110 | 110 | 110 |
| Observations | 3190 | 3190 | 3190 | 3190 | 3190 | 3190 |
| R^2^ | 0.059 | 0.021 | 0.088 | 0.007 | 0.296 | 0.063 |
| F Statistic | 48.562*** | 16.045*** | 74.291*** | 5.751*** | 1.545*** | 50.847*** |
| *Notes*: This table is the detailed version of Table 3 in the main text, with control variables shared. It presents the estimated relationship between economic uncertainty and the age-standardized prevalence of anxiety disorders, major depressive disorder, and eating disorders, specifically analyzing data corresponding to females. The primary variable of interest, economic uncertainty, is measured by the World Uncertainty Index (WUI). Country-level clustered robust standard errors are presented under parenthesis. Country-fixed effects (Country FE) are accounted for in all models, and year-fixed effects (Year FE) are incorporated in Columns (2), (4), and (6). The reported R-squared (R^2^) values correspond to the within R-squared for our fixed effects panel data models. The table presents robust standard errors under parenthesis, calculated using the Arellano robust variance estimator with country-level clustering to mitigate within-group error correlation. *p<0.1; **p<0.05; ***p<0.01 | | | | | | |

**Table A5** - The association between economic uncertainty and anxiety disorders, major depressive disorder, and eating disorders prevalence rates among males in detail.

|  | Dependent variables (Prevalence per 100,000 persons in a country) | | | | | |
| --- | --- | --- | --- | --- | --- | --- |
|  | Anxiety disorders | | Major depressive disorder | | Eating disorders | |
| Variables | (1) | (2) | (3) | (4) | (5) | (6) |
| WUI | 5.125*** | 2.774** | 1.800 | 2.707* | 0.489*** | -0.112 |
|  | (1.252) | (1.229) | (1.640) | (1.543) | (0.129) | (0.120) |
| Income | 27.292** | -18.039 | -41.319*** | -22.224 | 10.915*** | 4.139*** |
|  | (12.430) | (15.772) | (12.883) | (19.119) | (1.743) | (1.424) |
| Unemployement | 5.653** | 6.140** | -1.530 | -1.549 | -0.270 | -0.186 |
|  | (2.628) | (2.660) | (3.133) | (3.124) | (0.253) | (0.245) |
| Population | 78.318*** | -1.327 | -34.065 | -10.423 | -1.551 | -17.694** |
|  | (25.553) | (35.050) | (44.531) | (61.284) | (5.687) | (8.657) |
|  |  |  |  |  |  |  |
| Country FE | YES | YES | YES | YES | YES | YES |
| Year FE | NO | YES | NO | YES | NO | YES |
| # of Countries | 110 | 110 | 110 | 110 | 110 | 110 |
| Observations | 3190 | 3190 | 3190 | 3190 | 3190 | 3190 |
| R^2^ | 0.108 | 0.024 | 0.050 | 0.008 | 0.298 | 0.058 |
| F Statistic | 93.175*** | 18.462*** | 40.448*** | 5.979*** | 326.183*** | 47.242*** |
| *Notes*: This table is the detailed version of Table 4 in the main text, with control variables shared. It presents the estimated relationship between economic uncertainty and the age-standardized prevalence of anxiety disorders, major depressive disorder, and eating disorders, specifically analyzing data corresponding to males. The primary variable of interest, economic uncertainty, is measured by the World Uncertainty Index (WUI). Country-level clustered robust standard errors are presented under parenthesis. Country-fixed effects (Country FE) are accounted for in all models, and year-fixed effects (Year FE) are incorporated in Columns (2), (4), and (6). The reported R-squared (R^2^) values correspond to the within R-squared for our fixed effects panel data models. The table presents robust standard errors under parenthesis, calculated using the Arellano robust variance estimator with country-level clustering to mitigate within-group error correlation. *p<0.1; **p<0.05; ***p<0.01 | | | | | | |

**Table A6** - Economic uncertainty and anxiety disorders prevalence: Age-specific analysis.

|  | Dependent variables: Anxiety disorders prevalence (Prevalence per 100,000 persons in a country) | | | | | | | | | | | | |
| --- | --- | --- | --- | --- | --- | --- | --- | --- | --- | --- | --- | --- | --- |
|  | 15-19 ages | 20-24 ages | 25-29 ages | 30-34 ages | 35-39 ages | 40-44 ages | 45-49 ages | 50-54 ages | 55-59 ages | 60-64 ages | 65-69 ages | 70-74 ages | 75+ ages |
| Variables | (1) | (2) | (3) | (4) | (5) | (6) | (7) | (8) | (9) | (10) | (11) | (12) | (13) |
| WUI | 4.230* | 6.727^***^ | 7.257^**^ | 6.718^**^ | 4.974^*^ | 3.514 | 3.029 | 3.096 | 3.355^*^ | 3.555^*^ | 3.566^*^ | 3.485^*^ | 2.683^*^ |
|  | (2.535) | (2.536) | (2.864) | (2.841) | (2.603) | (2.379) | (2.144) | (1.905) | (1.843) | (1.964) | (2.007) | (1.857) | (1.408) |
| Income | -11.238 | -30.495 | -28.757 | -24.299 | -11.866 | 1.233 | 1.874 | -9.329 | -24.843 | -33.321 | -34.678 | -29.793 | -4.173 |
|  | (30.946) | (35.735) | (37.862) | (37.043) | (35.471) | (34.569) | (32.789) | (30.856) | (31.313) | (33.744) | (33.994) | (32.109) | (27.480) |
| Unemployement | 15.983** | 18.400^***^ | 18.369^***^ | 16.863^***^ | 14.754^**^ | 12.805^**^ | 11.344^**^ | 10.385^**^ | 9.476^**^ | 8.204^*^ | 6.532 | 4.794 | 2.916 |
|  | (6.979) | (6.307) | (6.653) | (6.470) | (6.016) | (5.629) | (5.222) | (4.780) | (4.600) | (4.883) | (4.997) | (4.566) | (3.291) |
| Population | 10.996 | -135.203 | -108.577 | -92.730 | -55.563 | -31.234 | -21.957 | -34.497 | -82.041 | -106.601 | -76.011 | -22.255 | -9.969 |
|  | (77.822) | (104.689) | (85.032) | (84.615) | (79.369) | (76.209) | (70.553) | (63.533) | (61.134) | (68.160) | (70.286) | (69.868) | (95.554) |
|  |  |  |  |  |  |  |  |  |  |  |  |  |  |
| Country FE | YES | YES | YES | YES | YES | YES | YES | YES | YES | YES | YES | YES | YES |
| Year FE | YES | YES | YES | YES | YES | YES | YES | YES | YES | YES | YES | YES | YES |
| # of Countries | 110 | 110 | 110 | 110 | 110 | 110 | 110 | 110 | 110 | 110 | 110 | 110 | 110 |
| Observations | 3190 | 3190 | 3190 | 3190 | 3190 | 3190 | 3190 | 3190 | 3190 | 3190 | 3190 | 3190 | 3190 |
| R^2^ | 0.028 | 0.038 | 0.029 | 0.022 | 0.015 | 0.010 | 0.009 | 0.010 | 0.015 | 0.019 | 0.016 | 0.011 | 0.005 |
| F Statistic | 22.059*** | 30.308^***^ | 22.804^***^ | 17.241^***^ | 11.407^***^ | 7.679^***^ | 6.679^***^ | 7.735^***^ | 11.484^***^ | 14.660^***^ | 12.249^***^ | 8.730^***^ | 4.070^***^ |
| *Notes*: This table presents information on anxiety disorders from Figure 4 in the main text. The coefficients from the fixed-effects model analyze the relationship between economic uncertainty and the age-standardized prevalence of major depressive disorders per 100,000 population in 110 countries. Each column corresponds to a specific age group, and the same model was applied to each. The primary variable of interest is economic uncertainty, represented by the World Uncertainty Index (WUI) in its current form. Control variables include the natural logarithm of GDP per capita as income, the unemployment rate, and the natural logarithm of the population. All estimations include country-fixed effects (Country FE) and year-fixed effects (Year FE). The reported R-squared (R^2^) values correspond to the within R-squared for our fixed effects panel data models. The table presents robust standard errors under parenthesis, calculated using the Arellano robust variance estimator with country-level clustering to mitigate within-group error correlation. *p<0.1; **p<0.05; ***p<0.01 | | | | | | | | | | | | | |

**Table A7** - Economic uncertainty and major depressive disorder prevalence: Age-specific analysis.

|  | Dependent variables: Major depressive disorder prevalence (Prevalence per 100,000 persons in a country) | | | | | | | | | | | | |
| --- | --- | --- | --- | --- | --- | --- | --- | --- | --- | --- | --- | --- | --- |
|  | 15-19 ages | 20-24 ages | 25-29 ages | 30-34 ages | 35-39 ages | 40-44 ages | 45-49 ages | 50-54 ages | 55-59 ages | 60-64 ages | 65-69 ages | 70-74 ages | 75+ ages |
| Variables | (1) | (2) | (3) | (4) | (5) | (6) | (7) | (8) | (9) | (10) | (11) | (12) | (13) |
| WUI | 0.654 | 2.326 | 3.386 | 4.590 | 5.403 | 6.280^*^ | 6.904^*^ | 7.127^**^ | 7.098^**^ | 6.973^*^ | 7.132^**^ | 7.648^**^ | 7.511^*^ |
|  | (2.100) | (3.297) | (3.126) | (3.164) | (3.494) | (3.775) | (3.571) | (3.467) | (3.540) | (3.586) | (3.480) | (3.670) | (4.448) |
| Income | -37.495 | -87.311^**^ | -72.579^*^ | -65.200 | -65.853 | -58.800 | -41.473 | -25.128 | -6.435 | 13.201 | 18.933 | 22.540 | 23.910 |
|  | (28.023) | (44.398) | (43.597) | (44.748) | (48.452) | (49.298) | (44.010) | (41.942) | (43.808) | (45.628) | (44.244) | (43.213) | (43.944) |
| Unemployement | -1.022 | -1.527 | 0.454 | 1.215 | -0.239 | -1.894 | -3.564 | -5.025 | -6.201 | -7.711 | -10.271 | -12.966^*^ | -12.683^*^ |
|  | (4.322) | (6.652) | (6.078) | (5.880) | (6.870) | (7.660) | (7.350) | (7.260) | (7.349) | (7.334) | (7.232) | (7.330) | (6.617) |
| Population | -105.223 | -127.278 | -106.973 | -109.987 | -115.075 | -103.725 | -59.337 | -14.523 | 19.320 | 86.829 | 177.788 | 265.238^*^ | 292.698^*^ |
|  | (96.161) | (134.723) | (125.069) | (131.534) | (146.780) | (153.320) | (138.742) | (129.589) | (130.712) | (134.554) | (141.431) | (147.067) | (154.858) |
|  |  |  |  |  |  |  |  |  |  |  |  |  |  |
| Country FE | YES | YES | YES | YES | YES | YES | YES | YES | YES | YES | YES | YES | YES |
| Year FE | YES | YES | YES | YES | YES | YES | YES | YES | YES | YES | YES | YES | YES |
| # of Countries | 110 | 110 | 110 | 110 | 110 | 110 | 110 | 110 | 110 | 110 | 110 | 110 | 110 |
| Observations | 3190 | 3190 | 3190 | 3190 | 3190 | 3190 | 3190 | 3190 | 3190 | 3190 | 3190 | 3190 | 3190 |
| R^2^ | 0.006 | 0.013 | 0.013 | 0.012 | 0.011 | 0.009 | 0.008 | 0.007 | 0.006 | 0.008 | 0.016 | 0.028 | 0.029 |
| F Statistic | 4.862^***^ | 10.382^***^ | 9.763^***^ | 9.623^***^ | 8.289^***^ | 6.900^***^ | 5.913^***^ | 5.198^***^ | 4.777^***^ | 6.282^***^ | 12.743^***^ | 21.951^***^ | 22.801^***^ |
| *Notes*: This table presents information on major depressive disorders from Figure 4 in the main text. The coefficients from the fixed-effects model analyze the relationship between economic uncertainty and the age-standardized prevalence of major depressive disorders per 100,000 population in 110 countries. Each column corresponds to a specific age group, and the same model was separately applied to each of them. The primary variable of interest is economic uncertainty, represented by the World Uncertainty Index (WUI) in its current form. Control variables include the natural logarithm of GDP per capita as income, the unemployment rate, and the natural logarithm of the population. All estimations include country-fixed effects (Country FE) and year-fixed effects (Year FE). The reported R-squared (R^2^) values correspond to the within R-squared for our fixed effects panel data models. The table presents robust standard errors under parenthesis, calculated using the Arellano robust variance estimator with country-level clustering to mitigate within-group error correlation. *p<0.1; **p<0.05; ***p<0.01 | | | | | | | | | | | | | |

**Table A8** - Economic uncertainty and eating disorders prevalence: Age-specific analysis.

|  | Dependent variables: Eating disorders prevalence (Prevalence per 100,000 persons in a country) | | | | | | |
| --- | --- | --- | --- | --- | --- | --- | --- |
|  | 15-19 ages | 20-24 ages | 25-29 ages | 30-34 ages | 35-39 ages | 40-44 ages | 45-49 ages |
| Variables | (1) | (2) | (3) | (4) | (5) | (6) | (7) |
| WUI | 0.042 | -0.180 | -0.276 | -0.270 | -0.228 | -0.107 | -0.043 |
|  | (0.387) | (0.511) | (0.513) | (0.469) | (0.350) | (0.215) | (0.132) |
| Income | 10.309^*^ | 10.500^*^ | 8.799 | 9.095^*^ | 7.159^*^ | 3.569 | 0.726 |
|  | (5.326) | (6.212) | (6.228) | (5.453) | (4.096) | (2.592) | (1.570) |
| Unemployement | -0.509 | -0.157 | -0.084 | -0.296 | -0.257 | -0.060 | -0.066 |
|  | (0.753) | (1.002) | (1.000) | (0.932) | (0.670) | (0.377) | (0.212) |
| Population | -56.937^***^ | -110.264^***^ | -106.199^***^ | -89.801^***^ | -68.501^***^ | -46.771^***^ | -31.632^***^ |
|  | (20.363) | (22.439) | (29.341) | (29.221) | (23.380) | (14.994) | (9.347) |
|  |  |  |  |  |  |  |  |
| Country FE | YES | YES | YES | YES | YES | YES | YES |
| Year FE | YES | YES | YES | YES | YES | YES | YES |
| # of Countries | 110 | 110 | 110 | 110 | 110 | 110 | 110 |
| Observations | 3190 | 3190 | 3190 | 3190 | 3190 | 3190 | 3190 |
| R^2^ | 0.061 | 0.090 | 0.080 | 0.074 | 0.085 | 0.111 | 0.141 |
| F Statistic | 49.183^***^ | 75.151^***^ | 65.959^***^ | 61.184^***^ | 70.630^***^ | 95.502^***^ | 124.896^***^ |
| *Notes*: This table presents information on eating disorders from Figure 4 in the main text. The coefficients from the fixed-effects model analyze the relationship between economic uncertainty and the age-standardized prevalence of major depressive disorders per 100,000 population in 110 countries. Each column corresponds to a specific age group, and the same model was separately applied to each of them. The primary variable of interest is economic uncertainty, represented by the World Uncertainty Index (WUI) in its current form. Control variables include the natural logarithm of GDP per capita as income, the unemployment rate, and the natural logarithm of the population. All estimations include country-fixed effects (Country FE) and year-fixed effects (Year FE). The reported R-squared (R^2^) values correspond to the within R-squared for our fixed effects panel data models. The table presents robust standard errors under parenthesis, calculated using the Arellano robust variance estimator with country-level clustering to mitigate within-group error correlation. *p<0.1; **p<0.05; ***p<0.01 | | | | | | | |
